# Consumption of Fructose by Cultured Primary Rat Astrocytes

**DOI:** 10.1007/s11064-026-04873-w

**Published:** 2026-08-27

**Authors:** Mariana Mesa-Leal, Ralf Dringen

**Affiliations:** 1 Faculty 2 (Biology/Chemistry), Centre for Biomolecular Interactions Bremen, P.O. Box 330440, 28334 Bremen, Germany; 2 Centre for Environmental Research and Sustainable Technologies, University of Bremen, Bremen, Germany

**Keywords:** Astrocytes, ATP, Fructose, Glycolysis, Metabolism, Respiration

## Abstract

Fructose has recently gained substantial interest in the context of diseases that are connected with sucrose-rich diets. Several studies have shown that astrocytes are able to metabolize fructose to some extent but less efficiently than glucose. To study whether and how fructose can fuel astrocytic energy metabolism, we used cultured primary astrocytes as model. These cells consumed fructose in a time- and concentration-dependent manner that followed apparent Michaelis-Menten type kinetics with a K_M_ value of 2.8 ± 0.9 mM and a V_Max_ value of 325 ± 32 nmol/(mg × h). Although the specific consumption of glucose and the production of lactate from glucose were around 4 times higher than the respective values for fructose, the loss of astrocytic cellular ATP during starvation was prevented by both fructose and glucose with similar concentration dependencies. However, severe differences were observed for fructose- and glucose-fed astrocytes, if the mitochondrial oxidative phosphorylation was impaired. For fructose-treated astrocytes the application of inhibitors of mitochondrial ATP regeneration almost doubled the low basal glycolytic lactate production, but this was insufficient to prevent a rapid and severe loss in cellular ATP content and subsequent cell toxicity. In contrast, for glucose-treated cells a strong upregulation of glycolysis was connected with a slow ATP loss and a delayed onset of toxicity. Although hexokinase in astrocytic cell lysates was found to have a high K_M_ value for fructose (4.4 ± 1.1 mM), hardly any free fructose was found in astrocytic lysates even after exposure to 10 mM fructose, suggesting that not the phosphorylation but rather the uptake of fructose limits astrocytic fructose consumption. The data presented demonstrate that millimolar concentrations of fructose are required to make this hexose a suitable extracellular substrate for astrocytic metabolism and that fructose metabolism provides energy mainly by mitochondrial respiration.

**Supplementary Information:**

The online version contains supplementary material available at https://doi.org/10.1007/s11064-026-04873-w.

## Introduction

Fructose is a ketohexose that is present as monosaccharide in fruits, honey, and other foods, but fructose is also a component of the disaccharide sucrose. Due to its sweetness, stability and solubility, fructose is frequently added to replace other sugars in manufactured products [1]. During the last decades the consumption of sucrose and high-fructose corn syrup has been drastically increased in manufactured foods and drinks. Adverse consequences of such a high fructose diet have been observed and connected with disorders such as obesity, diabetes, inflammation, cardiovascular abnormalities and cancer [2–6].

Dietary fructose is taken up by the cells of the small intestine via the GluT5 transporter [7, 8], phosphorylated in the cells by fructokinase (ketohexokinase) and metabolized to glucose and organic acids [9]. The efficiency of this intestinal fructose absorption depends on the source of fructose ingested. Intestinal uptake of free fructose, as derived from high-fructose corn syrup, relies mainly on the low-capacity GluT5 transporter and the intestinal absorptive capacity can become limited when large amounts of fructose are consumed [7, 10, 11]. If fructose is co-ingested with glucose, as typically occurs with fruits or sucrose-containing foods, fructose is absorbed more efficiently, an effect attributed to glucose-facilitated fructose uptake [10, 11]. Once absorbed, fructose metabolism is identical regardless of its dietary origin. The efficient intestinal fructose metabolism is overwhelmed by high doses of dietary fructose [9] resulting in fructose reaching the liver, which is considered to remove most of the remaining fructose [9]. Accordingly, the basal concentration of fructose in blood serum is typically within the low micromolar concentration range (5 µM [12]; 19 µM [13]; 46 µM [14]; 100 µM [15]). However, this low basal level was found strongly and rapidly increased after excessive fructose intake to values between 200 and 400 µM [12, 13], but recovers to normal concentrations within a few hours [12, 13]. An overconsumption of fructose has been connected with alterations in brain function, including neuroinflammation, mitochondrial disfunctions, alterations in synaptic plasticity and cognition as well as impairment in learning and memory [16–19].

Astrocytes are important partners of neurons and have many important functions in brain [20–25]. At least in culture, astrocytes have a broad metabolic potential and have been shown to take up and metabolize a wide range of exogenous molecules as energy substrates, including various hexoses, amino acids, monocarboxylates, and fatty acids [24, 26–29]. Fructose has also been reported to be metabolized by astrocytes and utilized as exogenous substrate to fuel various metabolic pathways (Table 1). The uptake of fructose in cultured astrocytes may involve GluT5 [37], but also diffusion has been discussed to mediate fructose uptake into astrocytes [38]. Fructose phosphorylation is most likely catalyzed in cultured astrocytes by hexokinase (HK), as such cultures do not contain immunocytochemically detectable fructokinase nor was activity of this enzyme measurable in astrocyte cultures [30]. Extracellular fructose has been reported to be used by cultured rat astrocytes as carbon source for the synthesis of glycogen, lactate and pyruvate, as electron sources for the reduction of peroxides and xenobiotics, and as exogenous substrate to maintain a high cellular ATP content during starvation (Table 1). However, even if fructose was applied in concentrations of at least 5 mM, this hexose is used by cultured astrocytes less efficiently than glucose to fuel most of the pathways investigated (Table 1).

**Table 1:** Literature data on the use of fructose as substrate for the metabolism of cultured rat astrocytes

Metabolic effect	Effects (% of respective glucose control)	[Fructose] applied (mM)	References
Glycogen restoration after starvation	50	20	[30]
Lactate release	32	10	[30]
	45	5	[31]
	23	5	[32]
	27	5	[33]
Pyruvate release	70	5	[33]
Support of cumene hydroperoxide clearance	50	5	[34]
Support of rapid GSH regeneration after oxidative stress	30	5	[34]
Menadione-mediated WST1 reduction	6	5	[35]
β-lapachone-mediated WST1 reduction	36	5	[36]
Maintenance of ATP during starvation	108	5	[27]

In order to investigate in more detail the astrocytic fructose metabolism and to explore why fructose is considered as poor substrate for astrocytic metabolism, we studied the potential of cultured astrocytes to consume fructose and to use it as substrate to fuel glycolysis and mitochondrial oxidative phosphorylation. The data obtained demonstrate that cultured astrocytes consume extracellular fructose in a time- and concentration-dependent manner and confirm that fructose is efficiently used by starved astrocytes as exogenous substrate to maintain a high ATP content. The function of fructose as energy substrate depends mainly on oxidative phosphorylation as fructose-fed astrocytes, in contrast to glucose-fed cells, cannot sufficiently accelerate glycolysis to compensate for a compromised ATP regeneration caused by an impairment of mitochondrial respiration, leading to rapid ATP depletion and subsequent cell death. In addition, we demonstrate that fructose uptake, rather than its phosphorylation by HK, predominantly limits fructose catabolism in astrocytes.

## Materials and Methods

### Materials

Sterile cell culture consumables and non-sterile 96-well plates were obtained from Sarstedt (Nümbrecht, Germany). Bovine serum albumin, dimethyl sulfoxide (DMSO), NAD^+^, NADP^+^, and NADPH were purchased from Applichem (Darmstadt, Germany). Adenosine triphosphate (ATP), glucose-6-phosphate dehydrogenase (G6PDH), glutamate-pyruvate transaminase (GPT), hexokinase (HK), lactate dehydrogenase (LDH), and hexose phosphate isomerase (HPI) were obtained from Roche Diagnostics (Mannheim, Germany). D-Glucose, D-fructose, BAM15, antimycin A, oligomycin A, and 2-deoxy-D-glucose (2DG) were purchased from Sigma-Aldrich (Steinheim, Germany). Dulbecco’s modified Eagle’s Medium (DMEM, containing 25 mM glucose; catalog number: 52100039), fetal calf serum (FCS) and penicillin G/streptomycin sulfate solution were purchased from Thermo Fisher Scientific (Schwerte, Germany). G6PDi-1 was from Cayman Chemical (Tallin, Estonia). The GluT5 inhibitor N-[4-(methylsulfonyl)−2-nitrophenyl]−1,3-benzodioxol-5-amine (MSNBA) was purchased from MedChem Express (Monmouth Junction, USA). The Cell Titer Glo^®^ 2.0 ATP Assay Kit was from Promega (Walldorf, Germany). All other basic chemicals were acquired from Sigma-Aldrich (Steinheim, Germany), Roth (Karlsruhe, Germany), or Merck (Darmstadt, Germany).

### Astrocyte-Rich Primary Cultures

Astrocyte-rich primary cultures were prepared from the total brains of neonatal Wistar rats, following the method described previously in detail [39]. The rats had been purchased from Charles River Laboratories (Sulzfeld, Germany) and they were treated in accordance to the State of Bremen, German, and European animal welfare acts. The isolated viable brain cells were seeded at a density of 300,000 cells per well in wells of 24-well plates (for experiments investigating hexose metabolism) and of 3,000,000 viable cells in 50 mm dishes (for cell lysates used for the analysis of HK kinetics) in 1 mL and 5 mL culture medium, respectively. The culture medium contained 90% DMEM (supplemented with 44.6 mM sodium bicarbonate, 1 mM pyruvate, 20 U/mL penicillin G, 20 µg/mL streptomycin sulphate) and 10% FCS. Without any further purification step, the cultures were maintained in a humidified atmosphere of a Sanyo (Osaka, Japan) CO_2_ incubator containing 10% CO_2_. The culture medium was renewed weekly and also on the day before conducting an experiment. For this study, astrocyte cultures of an age between 14 and 28 days were used. The cultures are characterized by a high prevalence of glial fibrillary acidic protein (GFAP)-positive astrocytes, with only low numbers of other brain cell types, including some microglia, oligodendrocytes, and ependymal cells [39–41].

### Experimental Incubation of Cultured Astrocytes

Confluent primary astrocyte cultures in wells of 24-well plates were washed twice with 1 mL of pre-warmed (37 °C) hexose-free incubation buffer (IB) (145 mM NaCl, 20 mM HEPES, 5.4 mM KCl, 1.8 mM CaCl_2_, 1 mM MgCl_2_, and 0.8 mM Na_2_HPO_4_, pH adjusted to 7.4 with 5 mM NaOH at 37 °C), and subsequently incubated in 200 µL of IB containing hexoses and/or inhibitors in the concentrations and for the durations specified in the legends of the figures and tables. All incubations were done at 37 °C in a humidified incubator (Sanyo, Japan) without CO_2_ supply. If one of the substances applied had been dissolved as stock solution in DMSO, control incubations were done with the appropriate final concentration of DMSO (up to 1%). This concentration of DMSO in the incubation medium did not affect cell viability nor alter hexose consumption compared to incubations without DMSO (Fig. S1). After a given incubation, the incubation media were collected for quantification of the extracellular concentrations of hexoses and lactate as well as the extracellular LDH activities. The cells were washed twice with 1 mL of ice-cold phosphate-buffered saline (PBS; 10 mM potassium phosphate buffer pH 7.4, containing 150 mM NaCl) and lysed for quantification of cellular contents of free hexoses or ATP.

### Determination of Extracellular Hexose Concentrations

The concentration of extracellular glucose was quantified by a coupled enzymatic test with HK and G6PDH as previously described in detail [39]. For the quantification of extracellular fructose, the glucose quantification assay was modified by the addition of HPI. In the assay, fructose becomes phosphorylated by hexokinase to fructose-6-phosphate that is isomerized to glucose-6-phosphate which is subsequently oxidized by G6PDH to yield 6-phosphogluconate and NADPH. After a given incubation period, incubation media samples were harvested, and when necessary, diluted beforehand to ensure reliable measurement. Aliquots of 10 µL of the media sample were diluted with 170 µL of pure water in a well of a microtiter plate before 180 µL of a freshly prepared reaction mixture was added to each well containing 0.3 M triethanolamine hydrochloride (TEA)/KOH buffer (pH 7.6), 2 mM MgCl_2_, 2 mM ATP, 1.5 mM NADP^+^, 0.3 U/well HK, 0.44 U/well G6PDH and 0.35 U/well HPI. After 30 min at room temperature, the absorbance of the formed NADPH was quantified at 340 nm and used to calculate the fructose concentration present in the incubation medium. For initial fructose concentrations up to 10 mM the reaction in a HPI-containing reaction mixture was completed within 30 min and was proportional to the applied fructose concentration (Fig. S2). In the absence of HPI, fructose did not cause any increase in absorbance, while presence of glucose was rapidly detected (Fig. S2).

### Determination of the Cellular Hexose Contents

Cellular levels of fructose and glucose were quantified in neutralized perchloric acid lysates of cultured astrocytes using a modified version of the method recently published [42], following the assay principle described above for determining the extracellular hexoses. The generated NADPH was quantified via its fluorescence signals using appropriate glucose standards. After a given incubation, the cells in wells of 24-well dishes were washed twice with 1 mL of ice-cold PBS before lysing in 200 µL of ice-cold 0.5 M HClO_4_ for 5 min on ice. The entire volume of the lysate was collected and 57 µL of ice-cold 2 M KOH was added for neutralization. Glucose standards in HClO_4_ (0–40 nmol/200 µL) were treated identically. The precipitating KClO_4_ was removed by centrifugation (5 min at 12,100*g*) and 120 µL of the supernatant were mixed in a well of a black microtiter plate with 60 µL of 0.3 M TEA/KOH buffer. The enzymatic reaction was initiated by adding 180 µL of the reaction mixtures used for quantification of either extracellular fructose or glucose. After 30 min at room temperature, the fluorescence of the generated NADPH was measured in a Fluoroskan Ascent FL microtiter plate reader (Thermo Fisher Scientific, Bremen, Germany) by using the 355 nm excitation filter and the 460 nm emission filter. The contents of cellular hexoses were calculated by making use of the results of the linear fluorescence calibration curve obtained by the standards. To obtain the specific cellular hexose contents, the determined cellular hexose values were normalized on the protein content of the respective culture.

### Determination of Extracellular Lactate

Extracellular lactate that had been released from the cells during the incubation was determined by a coupled-enzymatic assay with LDH and GPT as previously described [39]. The absorption at 340 nm of the NADH generated during the enzymatic reaction was used to calculate the extracellular lactate concentration.

### Determination of Hexokinase Activity

HK activity was measured in lysates obtained from cultured astrocytes in 50 mm dishes by a modification of the procedure previously reported [43, 44]. The cells were washed twice with 4 mL of ice-cold PBS, followed by a brief rinse with 2 mL 20 mM HEPES/NaOH (pH 7.3) on ice before they were lysed in 2 mL lysis buffer (20 mM HEPES/NaOH pH 7.3 containing 0.1% (v/v) Triton X-100) for 10 min on ice. The lysate was collected and centrifuged (5 min at 12,100 g, 4 °C) and the supernatants from several dishes were harvested and combined for measuring HK activity and soluble protein content [44]. HK activity in presence of glucose or fructose was measured by coupled enzymatic assays that use the G6PDH-mediated NADPH formation as indicator reaction. Glucose or fructose solutions (80 µL) containing different concentrations of the hexoses (dissolved in water) were mixed with 180 µL of the reaction mixture before 100 µL lysate supernatant were added to start the reaction. Final concentrations of the components of the glucose-containing reaction (360 µL) were 100 mM HEPES/NaOH buffer pH 7.3, 1 mM ATP, 2 mM MgSO_4_, 0.75 mM NADP^+^, and 1 U/well G6PDH. For measuring HK activity in the presence of fructose the reaction mixture contained additionally HPI (0.35 U/well). HK activity was monitored by measuring the increase in NADPH absorbance at 340 nm at room temperature for up to 20 min [44].

### Determination of Cellular ATP and Protein Contents and Assessment of Cell Viability

Cellular ATP contents were determined in neutralized perchlorate lysates as recently described in detail [26, 27, 45] by a luciferine-luciferase-based luminometric assay using the Cell Titer Glo® 2.0 ATP Assay Kit. To obtain the specific cellular ATP content, the determined ATP values were normalized on the protein content of the respective culture. The protein content of astrocyte cultures was quantified using the Lowry assay [46], with bovine serum albumin as the standard. Potential cytotoxicity of the treatments was evaluated by testing for the release of the cytosolic enzyme LDH. Extracellular LDH activity was measured in 10 µL aliquot parts of incubation media and compared to the initial cellular LDH activity (determined after total lysis of the cells in 1% (v/v) Triton X-100) [39].

### Statistical Analysis

The data shown are expressed as means ± standard deviation (SD) of values from three or more independent experiments conducted in duplicates or triplicates (for initial values). Normal distribution was confirmed by the Kolomogorov-Smirnov test for data sets derived from more than 5 independent experiments, while normal distribution of data was assumed for data sets derived from 5 or less independent experiments. Statistical significance between groups was assessed using one-way Analysis of Variance (ANOVA) followed by the Bonferroni post-hoc test as indicated by asterisks (*), if not stated otherwise. For comparisons between two data sets, a paired, one-tailed t-test was applied (indicated by hashtags, #). The significance of differences compared to the respective control is indicated by ^#/*^p < 0.05, ^##/**^p < 0.01, and ^###/***^p < 0.001. *p* > 0.05 was considered as not significant.

## Results

### Fructose Consumption by Cultured Astrocytes

**Fig. 1:**
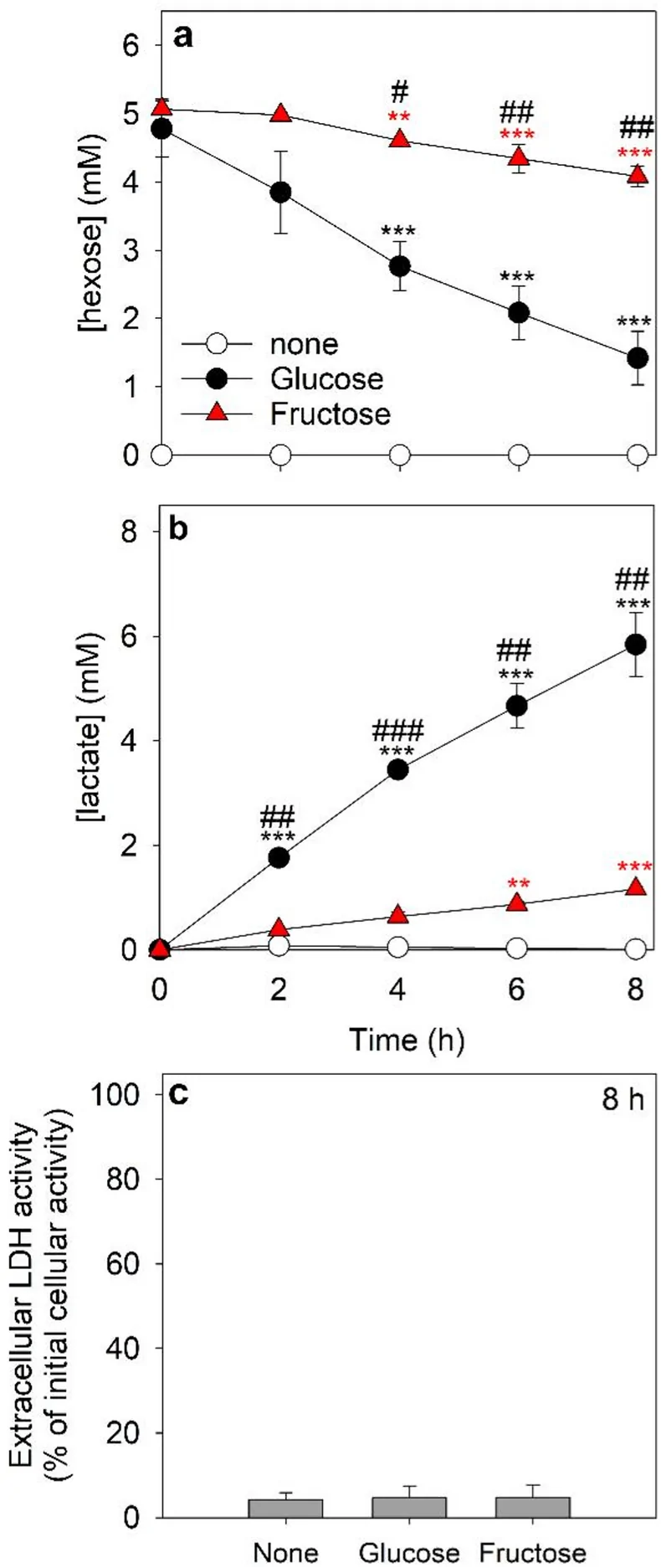
Time-dependent consumption of fructose and glucose by cultured astrocytes. Cultured primary astrocytes were incubated for the given incubation times in the absence of hexoses (none) or in the presence of either 5 mM fructose or 5 mM glucose. The extracellular concentrations of fructose or glucose (**a**), the extracellular lactate concentrations (**b**) and the extracellular LDH activities (**c**) were determined for the given incubation periods. The data presented are means ± SD of values from three individual experiments that had been performed on independently prepared cultures. The initial LDH activity was 118 ± 5 nmol/(min × well) and the initial protein content was 135 ± 12 µg/well. The significance of differences (ANOVA with Bonferroni post hoc test) of data compared to the values obtained for the initial time point are indicated in the colors of the respective symbols by *p < 0.05, **p < 0.01, and ***p < 0.001. The significance of differences (t-test) compared to the values obtained for fructose- and glucose-fed cells is indicated by ^#^*p* < 0.05, ^##^*p* < 0.01, and ^###^*p* < 0.001

To investigate the ability of astrocytes to metabolize fructose, primary cultured astrocytes were incubated in glucose-free IB that had been supplemented with 5 mM fructose. The applied extracellular fructose concentration declined over the time with a slow but consistent rate (Fig. 1a) of 0.204 ± 0.019 µmol/(mg x h), reaching after 24 h detectable concentrations of around 2.5 mM (Tables 2 and 3). In contrast, consumption of glucose by cultured astrocytes was much faster (Fig. 1a) with an initial rate of 0.780 ± 0.059 µmol/(mg x h). Within 24 h the initially applied concentration of glucose (5 mM) had been lowered by the cells to 0.6 mM (Table 2). During incubation in the absence of fructose and glucose, no extracellular hexoses were detectable (Fig. 1a).

**Table 2:** Carbon consumption by astrocytes within 24 h after exposure to 5 mM fructose or glucose

Parameter determined	Incubation with 5 mM fructose		Incubation with 5 mM glucose	
	[Substance] (mM)	[Total carbon] (mM)	[Substance] (mM)	[Total carbon] (mM)
Detectable [hexose] after 24 h	2.062 ± 0.512^###^	12.373 ± 3.070^###^	0.583 ± 0.493	3.499 ± 2.961
Detectable [lactate] after 24 h	3.325 ± 0.732^###^	9.976 ± 2.195^###^	5.729 ± 0.661	17.188 ± 1.984
Detectable [carbon] as hexose plus lactate after 24 h	--	22.349 ± 2.048^#^	--	20.687 ± 2.351
Consumed carbon in 24 h	--	7.703 ± 2.010^#^	--	9.604 ± 1.867

Cultured primary astrocytes were incubated for 24 h in glucose-free incubation buffer that had been supplemented with either 5 mM of fructose or of glucose before the remaining extracellular hexose concentrations and the extracellular lactate concentrations were determined. These values are given as concentration of the respective substances as well as as concentration of carbon atoms that are contained in the respective substances. The initial cellular LDH activity was 112 ± 19 nmol/(min × well) and the initial protein content was 129 ± 13 µg/well. The extracellular LDH activities after the 24 h incubation with fructose and glucose accounted for 6 ± 3% and 11 ± 4% of the initial cellular LDH activity, respectively. The data shown are means ± SD of values from 9 independent experiments that had been performed on independently prepared cultures. The significance of differences (t-test) of data obtained for incubations with fructose and glucose are indicated by ^#^*p* < 0.05, ^##^*p* < 0.01, and ^###^*p* < 0.001

**Table 3:** Fructose consumption and fructose-dependent lactate release from cultured astrocytes after application of 5 mM fructose

Parameter investigated		Incubation for 4 h	Number of experiments (independent cultures)	Incubation for 24 h	Number of experiments (independent cultures)
[Fructose] detectable	(mM)	4.47 ± 0.22	16 (15)	2.50 ± 0.79	21 (16)
Fructose consumed	(mM)	0.79 ± 0.36	16 (15)	2.72 ± 0.90	21 (16)
Fructose consumed	(µmol/mg)	1.10 ± 0.51	16 (15)	4.01 ± 1.49	21 (16)
Lactate released	(mM)	0.98 ± 0.45	16 (15)	2.82 ± 0.89	21 (16)
Lactate released	(µmol/mg)	1.36 ± 0.45	16 (15)	4.21 ± 1.62	21 (16)
Lactate release/fructose consumed		1.59 ± 1.29	16 (15)	1.07 ± 0.20	21 (16)
Initial protein content	(µg/well)	144 ± 19	16 (15)	143 ± 33	21 (16)
Initial cellular LDH activity	(nmol/(min x well))	123 ± 18	16 (15)	127 ± 24	21 (16)
Extracellular LDH activity	(% of initial)	4 ± 2	16 (15)	5 ± 2	21 (16)

Primary astrocytes were exposed to 5 mM fructose and incubated for 4 h or for 24 h before the remaining extracellular concentrations of fructose, the extracellular concentrations of fructose-derived lactate as well as the extracellular LDH activities (in % of the initial cellular LDH activity) were measured. In addition, the initial protein contents per well and the initial cellular LDH activities of the cultures were determined and the specific fructose consumption, the specific lactate formation and the ratio of lactate released to fructose consumed were calculated. The results shown are means ± SD of values from a total of n independent experiments that had been performed on the given numbers of independently prepared cultures (numbers in brackets)

After exposure of cultured astrocytes to 5 mM fructose, lactate was produced (Fig. 1b) with an initial rate of 0.230 ± 0.019 µmol/(mg x h) that lead to an extracellular lactate accumulation of around 3 mM within 24 h of incubation (Tables 2 and 3). Compared to fructose-fed astrocytes, extracellular lactate accumulation was much faster during incubations with glucose (Fig. 1b) as indicated by an almost 5 times higher initial lactate accumulation rate (1.169 ± 0.197 µmol/(mg x h)), reaching around 6 mM within 8 h (Fig. 1b) which was not further altered during 24 h of incubation (Table 2). Hardly any lactate release was found for astrocytes that had been incubated without hexoses (Fig. 1b). None of the conditions applied caused any severe loss in cell viability as indicated by the absence of any substantial LDH release after incubations for 8 h (Fig. 1c) or 24 h (Table 3).

**Fig. 2:**
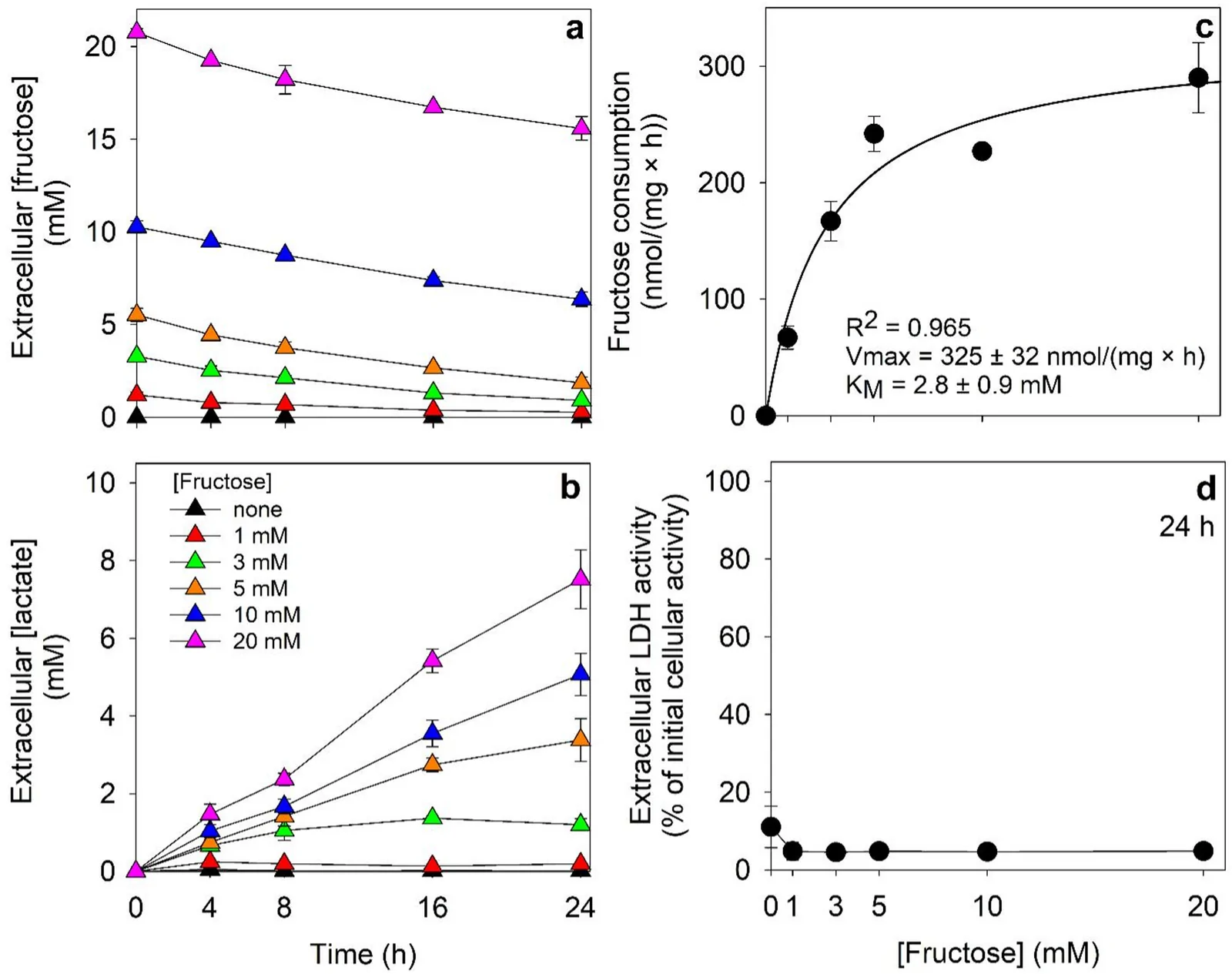
Concentration- and time-dependent consumption of fructose by cultured astrocytes. The cells were incubated for up to 24 h in glucose-free IB in the presence of fructose that had been applied in the given initial concentrations. The extracellular concentrations of fructose (**a**) and lactate (**b**) were determined for the incubation times indicated. The specific fructose consumption rates (**c**) were calculated from the initial linear decreases in the applied fructose concentrations for incubation of up to 16 h and the Vmax and K_M_ values (**c**) were calculated from these data. For 24 h incubations, the extracellular LDH activity was measured as an indicator for a potential loss in cell viability (**d**). The initial cellular LDH activity was 127 ± 25 nmol/(min × well) and the initial protein content was 143 ± 4 µg/well. The data represent the means ± SD of values from three independent experiments that had been performed on independently prepared cultures

To test for the concentration-dependency of the astrocytic fructose consumption, the cells were exposed to fructose in concentrations of up to 20 mM for up to 24 h. None of these conditions affected the cell viability, as indicated by the lack of any significant increase in extracellular LDH activity (Fig. 2d). A time-dependent decline in the extracellular fructose concentration was observed for all applied initial fructose concentrations (Fig. 2a). The specific fructose consumption rates (Fig. 2b) were calculated from the linear decline in extracellular fructose concentrations (Fig. 2a) and showed a hyperbolic relationship between the specific consumption rate and the initial fructose concentration applied (Fig. 2c). Fitting of these data by the Michaelis-Menten equation revealed that half-maximal fructose consumption occurred at an initial fructose concentration of 2.8 ± 0.9 mM ($\:{K}_{M}$) with a maximal consumption rate ($\:{V}_{Max}$) of 325 ± 32 nmol/(mg x h).

Fructose-treated astrocytes produced and released substantial amounts of lactate in a time- and concentration-dependent manner (Fig. 2b). For high concentrations of fructose applied (10 mM or 20 mM), the extracellular lactate concentration increased almost proportional with time throughout the complete incubation period of 24 h (Fig. 2b), while for lower fructose concentrations the rate of lactate production was slowed when most of the applied fructose had been consumed by the cells (Fig. 2a).

For most of the further experiments a fructose concentration of 5 mM was applied for incubation periods of 4 h or 24 h. Table 3 lists the data obtained for these conditions in a large number of independent experiments. During incubations for 4 h and 24 h, cultured astrocytes consumed on average 0.79 ± 0.36 mM (1.10 ± 0.51 µmol/mg) and 2.72 ± 0.90 mM (4.01 ± 1.49 µmol/mg) fructose, respectively, while the extracellular lactate had accumulated to 0.98 ± 0.45 mM (1.36 ± 0.45 µmol/mg) and 2.82 ± 0.89 mM (4.21 ± 1.62 µmol/mg) (Table 3). The absence of a substantial increase in extracellular LDH activity (Table 3) demonstrates that the treatment with fructose for up to 24 h had not compromised cell viability.

### Maintenance of Astrocytic ATP Contents by Fructose During Starvation

**Fig. 3:**
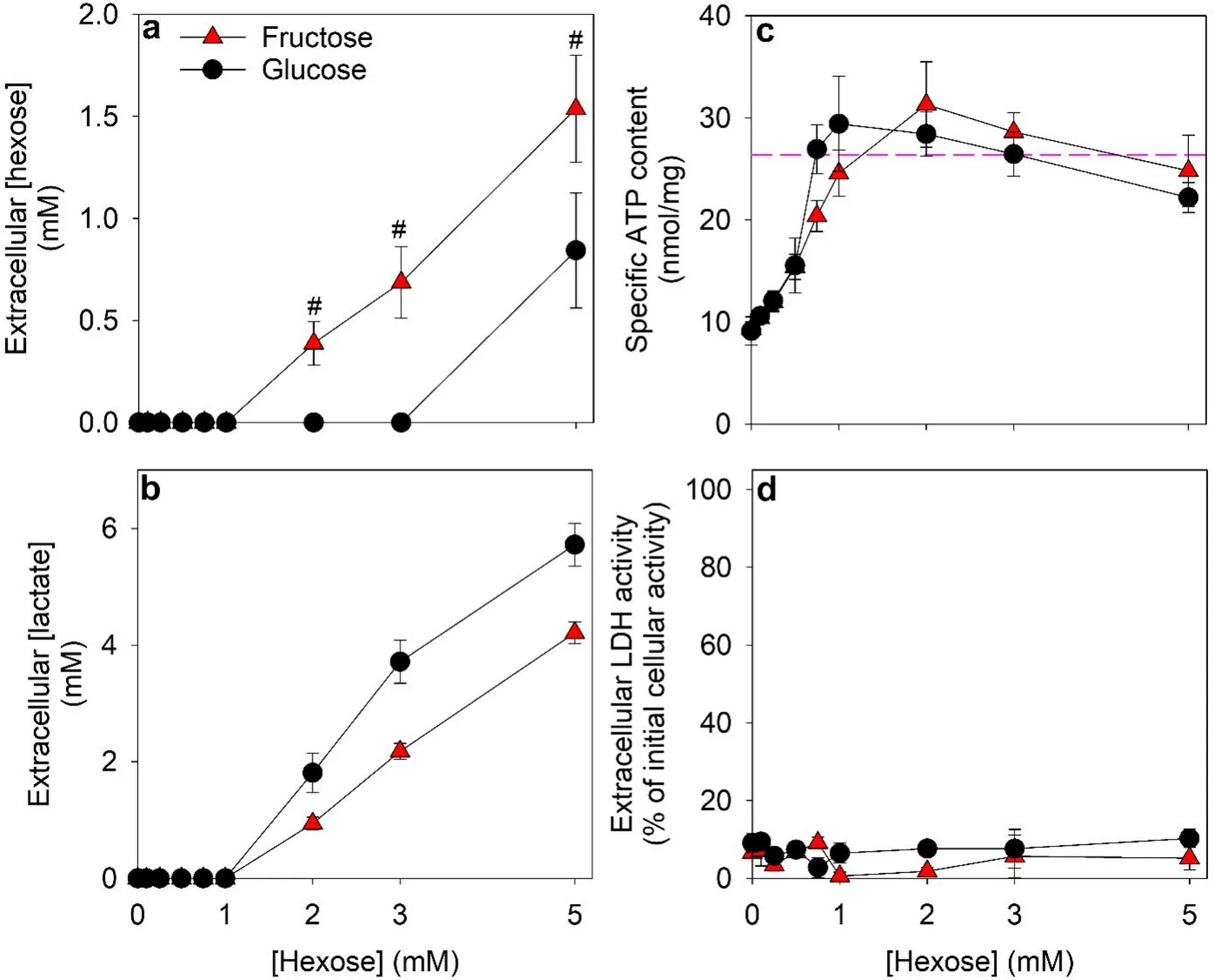
Use of fructose and glucose for ATP maintenance and lactate formation by cultured astrocytes. The cultures were incubated for 24 h with fructose or glucose in the given concentrations before the remaining extracellular hexose concentrations (**a**), the extracellular lactate concentrations (**b**), the specific cellular ATP contents (**c**) and the extracellular LDH activities (**d**) were determined. The initial cellular LDH activity was 107 ± 10 nmol/(min x well), the initial protein content was 129 ± 1 µg/well and the initial specific cellular ATP content was 26.4 ± 2.0 nmol/mg (dashed line in **c**). The data represent the means ± SD of values from three independent experiments that had been performed on independently prepared cultures. The significance of differences (t-test) of data obtained for incubations with a given initial concentration of fructose and glucose are indicated by ^#^*p* < 0.05, ^##^*p* < 0.01, and ^###^*p* < 0.001

Fructose in a concentration of 5 mM has been shown to prevent the depletion of astrocytic ATP during a 24 h starvation period [27]. To test for the concentration-dependency of this ATP maintenance, cultured astrocytes were incubated with fructose or glucose in concentrations of up to 5 mM. After 24 h of incubation with 1 mM (or less) of either fructose or glucose, the hexoses had been completely metabolized (Fig. 3a) and also hexose-derived lactate was not detectable anymore (Fig. 3b). Incubations of astrocytes with higher concentrations of the hexoses revealed a significantly faster ability of the cells to consume glucose and to produce lactate from glucose compared to the respective fructose treatments (Fig. 3a, b). However, for both fructose and glucose already an initial concentration of 1 mM was sufficient to prevent a loss in cellular ATP content during an incubation of 24 h. No significant differences were observed for the ATP-maintaining effects of fructose and glucose in the concentrations investigated (Fig. 3c). None of the conditions applied caused any obvious toxicity as demonstrated by the absence of any significant increase in extracellular LDH activity (Fig. 3d).

Astrocytes consumed glucose more efficiently that fructose, but released also much higher amounts of glucose-derived lactate compared to the respective fructose incubations (Figs. 1 and 3; Table 2). To analyze how much of the carbon that had initially been applied as hexose (5 mM fructose or glucose, corresponding to 30 mM of carbon atoms in the hexoses) may have been oxidized during a 24 h incubation, we determined the remaining hexoses and the hexose-derived lactate in the medium. Although the levels of detectable extracellular hexoses and hexose-derived lactate differed strongly between fructose- and glucose-fed astrocytes (*p* < 0.001), the differences in the overall amount of carbon detectable as extracellular hexose plus lactate after a 24 h of incubation was with around 20 mM rather similar for both hexoses and differed only slightly (*p* < 0.05) (Table 2).

### Effects of an Inhibition of Metabolic Pathways on Astrocytic Fructose And Glucose Consumption

**Fig. 4:**
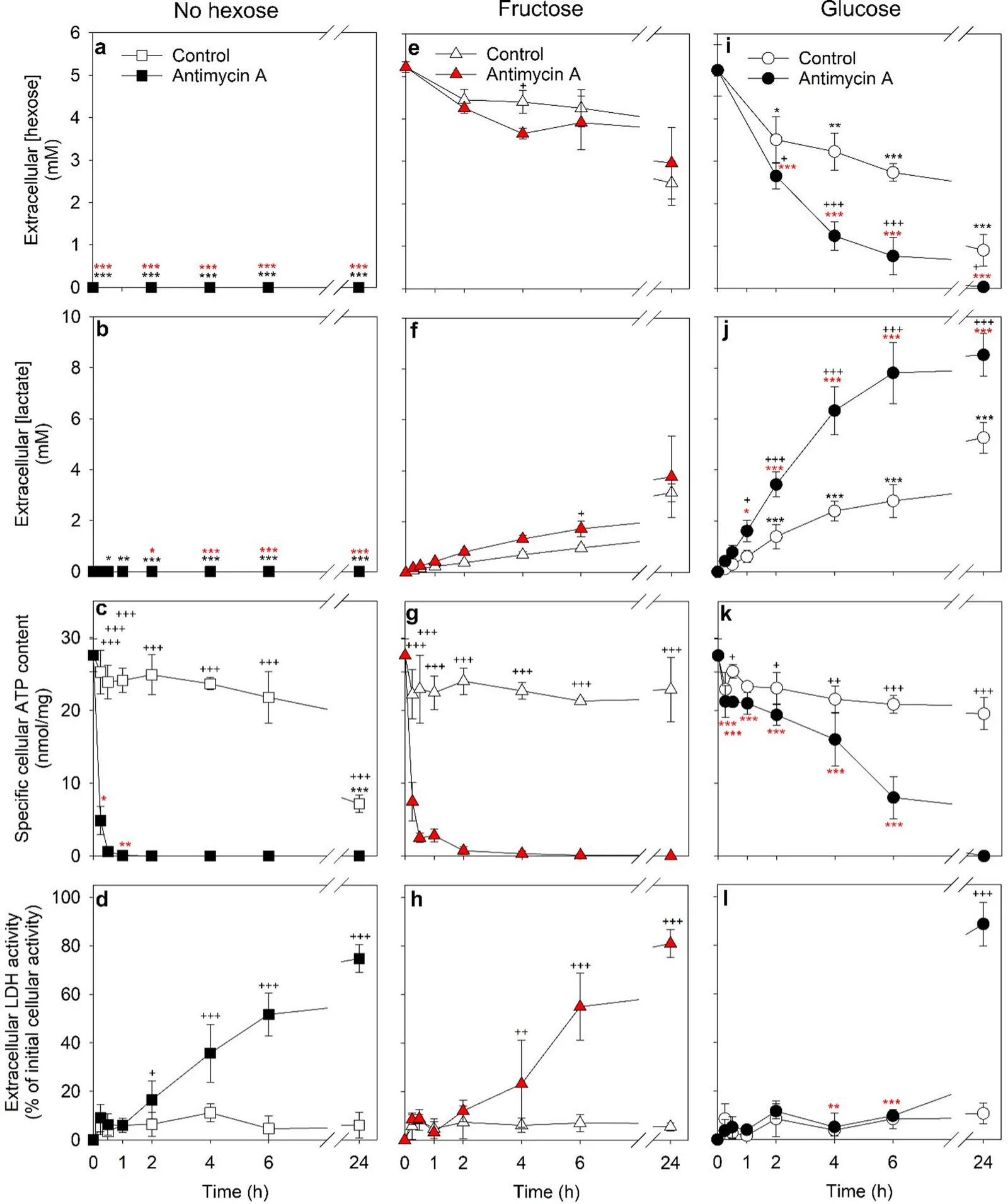
Effects of antimycin A on the fructose and glucose metabolism of cultured astrocytes. Cultured astrocytes were incubated for up to 24 h without hexose (**a**–**d**), with fructose (**e**–**h**; 5 mM initial concentration) or with glucose (**i**–**l**; 5 mM initial concentration) in the absence or the presence of antimycin A (1 µM). At the indicated time points, the extracellular concentrations of hexoses (**a**, **e**, **i**) and lactate (**b**, **f**, **j**), the specific cellular ATP contents (**c**, **g**, **k**), and the extracellular LDH activities (**d**, **h**, **l**) were determined. The initial cellular LDH activity was 112 ± 35 nmol/(min x well) and the initial protein content was 123 ± 21 µg/well. The data represent the means ± SD of values from three independent experiments that had been performed on independently prepared cultures. The significance of differences of data were calculated by two-way ANOVA, followed by Bonferroni post hoc test. Significant differences compared to the data obtained for the fructose treatment are indicated by asterisks by ^*^p < 0.05, ^**^p < 0.01, and ^***^p < 0.001 in black (control) or red (antimycin A), while the data obtained for incubations with and without antimycin A for a given substrate treatment is indicated by ^+^*p* < 0.05, ^++^*p* < 0.01, and ^+++^*p* < 0.001

Inhibition of mitochondrial respiration by antimycin A has been shown to accelerate glucose consumption and glucose-derived lactate production in cultured astrocytes [32, 47]. To test whether also fructose consumption is affected by the presence of antimycin A, cultured astrocytes were incubated without or with 5 mM of fructose or glucose in the absence or the presence of antimycin A for up to 24 h. During incubation in the absence of antimycin A (Fig. 4, open symbols), astrocytes consumed the applied hexoses and produced lactate from the metabolized hexoses (Fig. 4f, j) with rates similar to those shown in Fig. 1. In contrast, extracellular hexoses and lactate were not detected in astrocytes that had been incubated without hexoses (Fig. 4a, b). Quantification of the cellular ATP content for the incubations in the absence of antimycin A revealed that the specific cellular ATP contents declined only slowly during the incubation and accounted, after 6 h of incubation without or with hexoses, to around 80% of the initial values (Fig. 4c, g, k; open symbols). These values were maintained during the 24 h incubation period for astrocytes that had been exposed to either fructose (Fig. 4g) or glucose (Fig. 4k), while the specific ATP content of starved astrocytes (incubation without hexose) had been lowered to 25% of the initial content within 24 h (Fig. 4c). None of these antimycin A-free conditions caused any obvious toxicity as demonstrated by the absence of any significant increase in extracellular LDH activity (Fig. 4d, h, l; open symbols).

The presence of antimycin A severely affected astrocytes that had been incubated in the absence of hexoses (Fig. 4, filled symbols). The cellular ATP content disappeared almost completely within 30 min (Fig. 4c), in accordance to what has been previously observed [48], and the extracellular LDH activity as indicator of cell toxicity was significantly elevated already 2 h after onset of the incubation and increased strongly during longer incubations (Fig. 4d). The presence of fructose during the incubation with antimycin A did hardly slow the rapid loss in cellular ATP during the initial 30 min of incubation (Fig. 4g) and was unable to prevent the antimycin-induced toxicity (Fig. 4h), even if fructose had been applied in concentrations of up to 20 mM (Fig. S3). The fructose consumption (Fig. 4e) and the fructose-dependent lactate production (Fig. 4f) were significantly elevated by the presence of antimycin A. For glucose-treated astrocytes, the presence of antimycin A caused a strong increase in glucose consumption (Fig. 4i) and lactate production (Fig. 4j). In addition, the antimycin A-induced loss in cellular ATP found for cells that had been treated without hexoses or with fructose (Fig. 4c, g) was substantially delayed in glucose-fed astrocytes (Fig. 4k) and even after 6 h of incubation the specific ATP content accounted for around 30% of the initial content (Fig. 4k). In addition, no elevated LDH release was observed during the initial 6 h of incubation of antimycin A-exposed glucose-fed astrocytes, while after 24 h of incubation maximal LDH release was observed (Fig. 4l) accompanied with a complete absence of extracellular glucose (Fig. 4i).

**Fig. 5:**
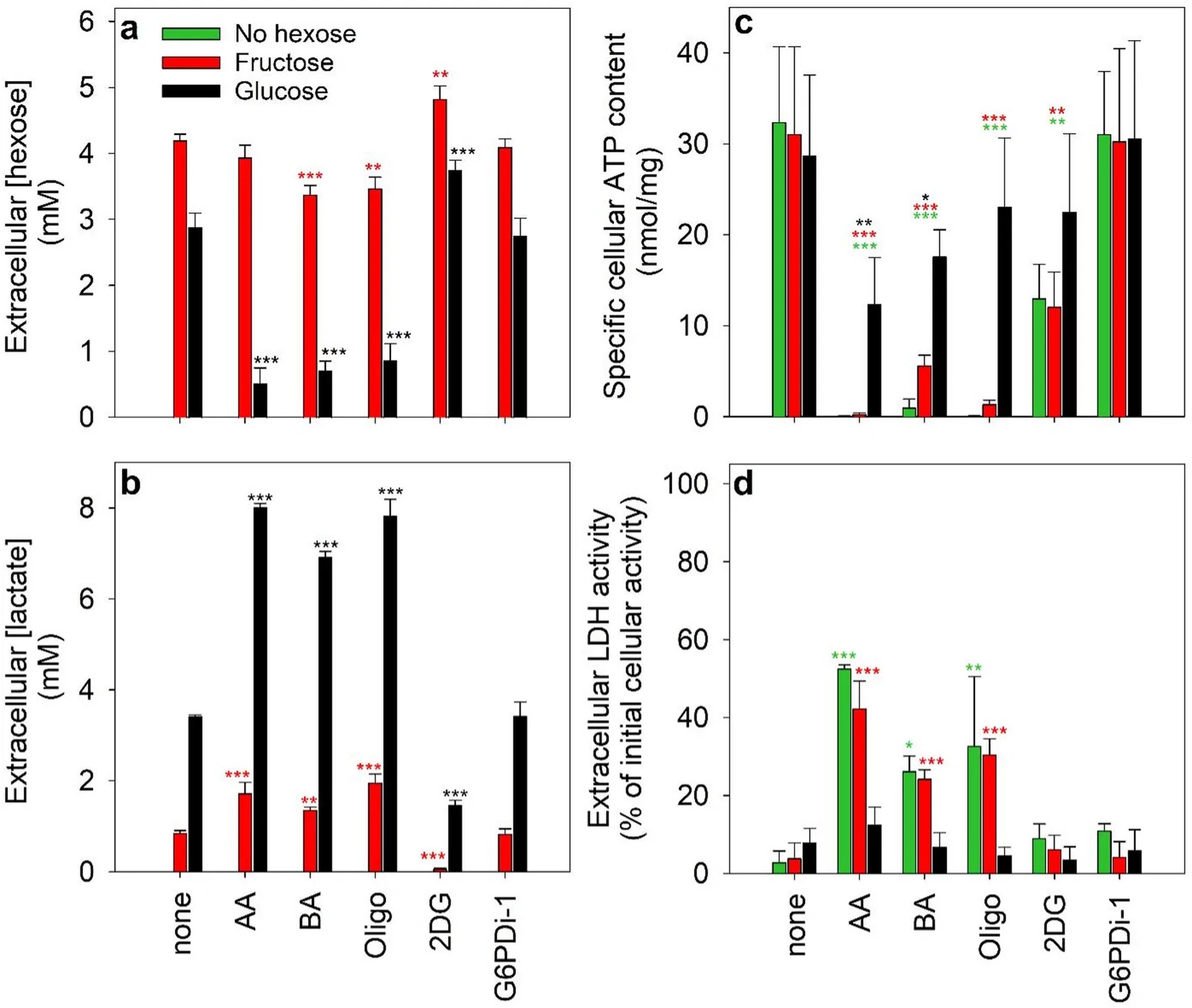
Effect of metabolic inhibitors and modulators on the consumption of fructose and glucose by astrocyte cultures. The cells were incubated without hexoses, with 5 mM fructose or with 5 mM glucose in the absence (none) or the presence of either 1 µM antimycin A (AA), 1 µM BAM15 (BA), 1 µM oligomycin A (Oligo), 5 mM 2DG or 10 µM G6PDi-1. After 6 h of incubation, the remaining concentrations of extracellular hexoses (**a**), the extracellular lactate concentrations (**b**), the specific cellular ATP contents (**c**) and the extracellular LDH activities (**d**) were determined. The initial cellular ATP content was 40.1 ± 10.2 nmol/mg, the initial cellular LDH activity was 103 ± 5 nmol/(min x well), and the initial protein content was 111 ± 19 µg/well. The data represent the means ± SD of values from three independent experiments that had been performed on independently prepared cultures. The significance of differences (ANOVA) compared with the respective control condition (none) is indicated in the colors used for each substrate condition with *p < 0.05, **p < 0.01, and ***p < 0.001

To further investigate the importance of metabolic pathways for the astrocytic utilization of fructose and glucose, cultured astrocytes were incubated for 6 h without or with 5 mM of either fructose or glucose in the absence or the presence of antimycin A, the uncoupler BAM15 [48], the ATP synthase inhibitor oligomycin A [49], the glycolysis inhibitor 2DG [42] or the pentose-phosphate pathway (PPP) inhibitor G6PDi-1 [50]. Compared to the respective control incubations (absence of inhibitors), the presence of antimycin A, BAM15 and oligomycin A accelerated to a low extend the consumption of fructose (Fig. 5a) and the production of lactate from fructose (Fig. 5b), but had stronger stimulating effects on the astrocytic glucose consumption (Fig. 5a) and lactate production from glucose (Fig. 5b). In astrocytes that had been incubated without hexoses or with fructose, hardly any cellular ATP was detectable after treatment with the mitochondrial inhibitors for 6 h (Fig. 5c), while the extracellular LDH activities were found severely increased (Fig. 5d). In contrast, the presence of glucose during the 6 h incubation with the mitochondrial inhibitors allowed the cells to maintain at least part of the cellular ATP content (Fig. 5c) and prevented the increase in extracellular LDH (Fig. 5d).

The presence of the glycolysis inhibitor 2DG significantly lowered the consumption of fructose and glucose by astrocytes (Fig. 5a) as well as the production of lactate from these hexoses (Fig. 5b). This treatment did only partially lower cellular ATP contents (Fig. 5c) and the cell viability was not compromised by the 2DG treatment (Fig. 5d). The presence of the PPP inhibitor G6PDi-1 did not affect any of the parameters investigated for incubations without or with either fructose or glucose (Fig. 5), suggesting that the PPP plays not a substantial role in the metabolism of both hexoses under the conditions studied.

### Test for the Effects of a GluT5 Inhibitor on Astrocytic Fructose Consumption

GluT5 is the transporter that is mainly responsible for cellular uptake of fructose [7, 8]. To test whether this transporter contributes to the observed consumption of fructose by cultured astrocytes, the potential of the specific GluT5 inhibitor MSNBA [51] to lower fructose consumption and lactate production from the applied fructose were investigated. During a 24 h incubation, fructose was consumed and lactate was produced from the fructose applied (Table 4). The presence of 100 µM of the GluT5 inhibitor MSNBA lowered at best partially the astrocytic fructose consumption and the fructose-derived lactate production (Table 4). The viability of the cells was not compromised by the conditions applied as demonstrated by the absence of any significant increase in extracellular LDH activity (Table 4).

**Table 4:** Effects of the GluT5 inhibitor MSNBA on the fructose consumption and fructose-dependent lactate generation by astrocytes

Parameter investigated		Control	MSNBA
[Fructose] detectable after 24 h	(mM)	3.07 ± 0.23	3.82 ± 0.43^#^
Fructose consumed in 24 h	(mM) (µmol/mg)	1.91 ± 0.32 3.75 ± 0.49	1.73 ± 0.43 3.40 ± 0.70
Lactate released in 24 h	(mM) (µmol/mg)	2.10 ± 0.32 4.19 ± 0.75	1.57 ± 0.26^#^ 3.12 ± 0.52^#^
Lactate released/fructose consumed		1.12 ± 0.16	0.93 ± 0.14^#^
Extracellular LDH activity	(%)	5 ± 1	8 ± 1^#^

Primary astrocytes were exposed to fructose in the initial concentration of 5 mM in the absence (control) or the presence of 100 µM of the GluT5 inhibitor MSNBA. After 24 h, the remaining extracellular concentrations of fructose, the extracellular concentrations of lactate as well as the extracellular LDH activities (in % of the initial cellular LDH activity) were measured and the ratio of lactate produced to fructose consumed was calculated. The initial cellular LDH activity was 119 ± 7 nmol/(min × well) and the initial protein content was 101 ± 9 µg/well. The results shown are means ± SD of values from three independent experiments that had been performed on independently prepared cultures. The significance of differences (t-test) compared to the values obtained for the control condition is indicated by ^#^*p* < 0.05

### Kinetic Parameters of Astrocytic Hexokinase for Fructose and Glucose

**Fig. 6:**
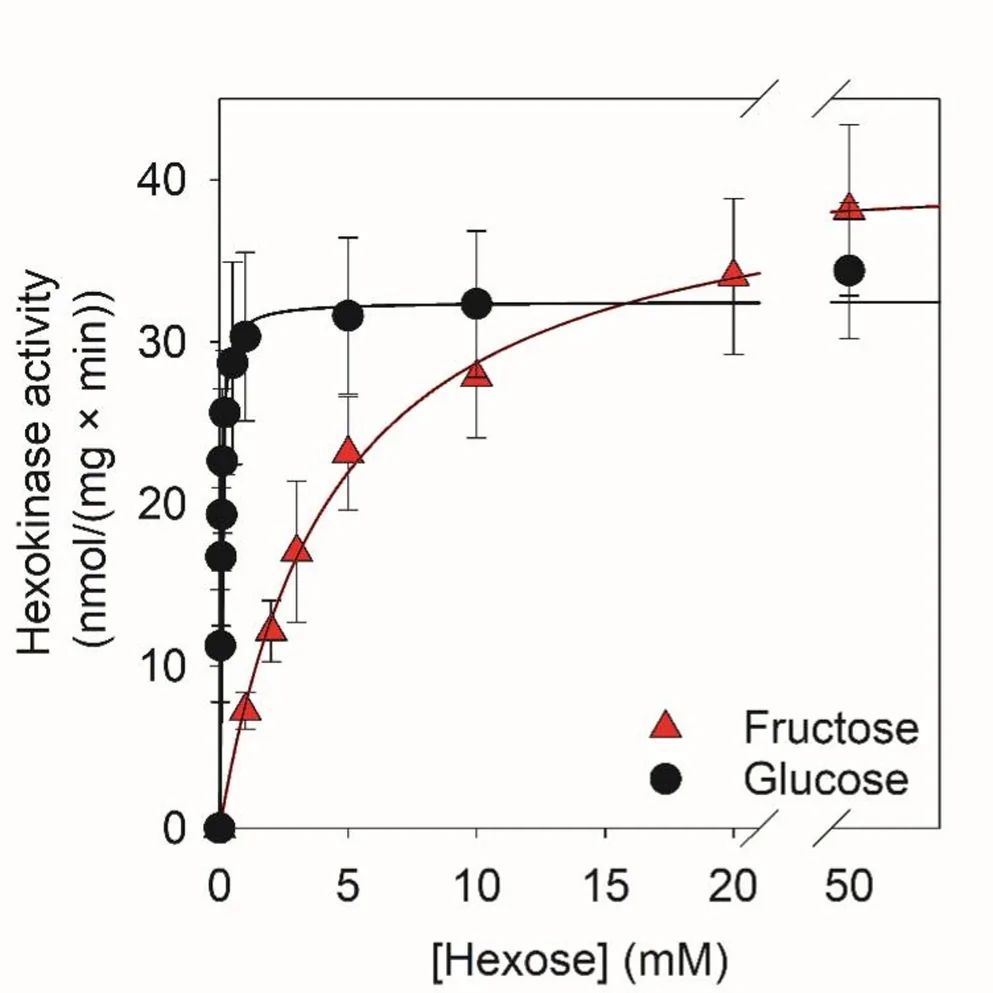
Phosphorylation of fructose and glucose by astrocytic hexokinase. The activity of HK in cell lysates from astrocyte-rich primary cultures was measured by coupled enzymatic assays for the indicated concentrations of fructose and glucose. The hyperbolic concentration-dependencies were used to calculate the kinetic parameters of HK for both hexoses (given in Table 5). The protein content of the lysates derived from 5 cm plates was 1089 ± 49 µg/dish. The data presented are means ± SD of values obtained in four experiments performed on lysate supernatants obtained from independently prepared astrocyte cultures

HK is considered as enzyme that is responsible for the phosphorylation of fructose in cultured astrocytes [30]. To determine the kinetic parameters of the HK present in cultured rat astrocytes for fructose (and glucose as a control), the HK activity in cell lysates was determined for different initial concentrations of the two hexoses (Fig. 6). For both fructose and glucose, hyperbolic Michaelis–Menten-type relationships were observed. While quite similar maximal specific HK activities were determined for fructose and glucose that did not significantly differ with 41 ± 12 nmol/(mg x min) for fructose and 32 ± 16 nmol/(mg x min) for glucose, the K_M_ values for the two hexoses differed by almost two orders of magnitude with 49 ± 10 µM for glucose and 4.4 ± 1.1 mM for fructose (Table 5).

**Table 5:** Kinetic parameters for the phosphorylation of fructose and glucose by the hexokinase in lysates of astrocyte cultures

Substrate	K_M_ (mM)	V_max_ (nmol/(mg x min))
Fructose	4.4 ± 1.1	41 ± 12
Glucose	0.049 ± 0.01	32 ± 16

The hyperbolic concentration-dependencies of HK activity for the substrates fructose and glucose (Fig. 6) were used to calculate the kinetic parameters of HK for the phosphorylation of fructose and glucose by fitting of the experimental data with the Michaelis-Menten equation. The data presented are means ± SD of values obtained in four experiments performed on lysates obtained from independently prepared astrocyte cultures

### Test for the Presence of Free Fructose or Glucose in Hexose-Exposed Cultured Astrocytes

**Fig. 7:**
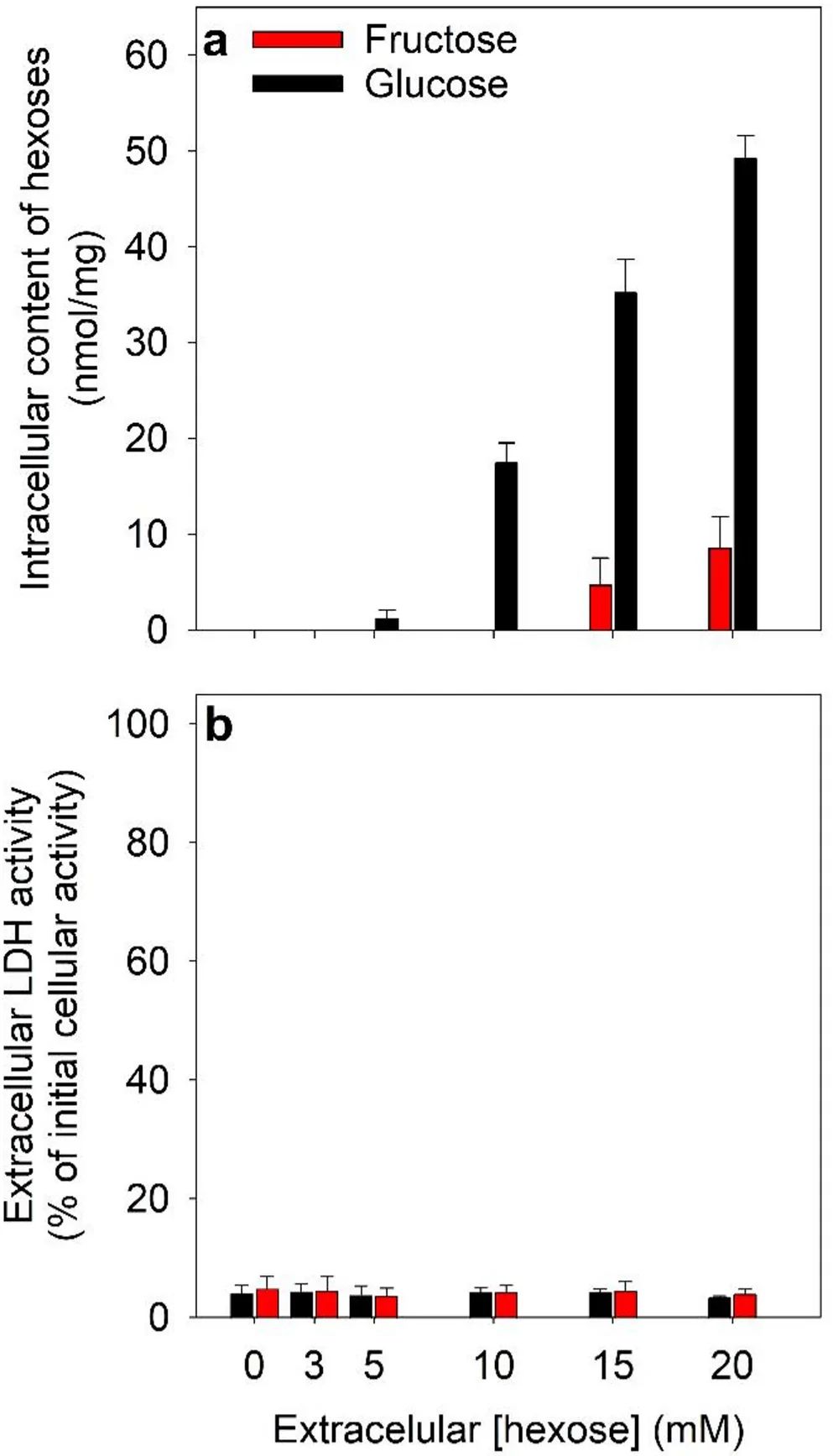
Cellular contents of free fructose and glucose in cultured astrocytes. The cells were incubated with the given concentrations of fructose or glucose for 1 h before the specific cellular contents of fructose and glucose (**a**) and the extracellular LDH activities (**b**) were determined. The initial cellular LDH activity was 119 ± 8 nmol/(min × well) and the initial protein content was 118 ± 18 µg/well. The data represent the means ± SD of values from three independent experiments that had been performed on independently prepared cultures

To test for the presence of unphosphorylated hexoses in the cells after exposure of cultured astrocytes to hexoses, the cells were incubated with fructose or glucose in concentrations of up to 20 mM for 1 h before the content of free hexoses were determined in the cell lysates. For fructose-treated astrocytes, free fructose was only found for incubations with 15 mM or 20 mM fructose in low specific levels of around 4.8 and 8.6 nmol/mg (Fig. 7). In contrast, substantial amounts of free glucose were found already after incubation with 10 mM glucose (17.5 ± 2.0 nmol/mg) and this level was found severely increase by 100% and 180% for cells that had been exposed to 15 mM and 20 mM glucose, respectively (Fig. 7). For hexose concentrations of 15 mM and 20 mM, the determined cellular levels of free glucose were around 7.4- and 5.7-times higher, respectively, than the levels of free fructose in cells that had been incubated with the respective concentrations of fructose (Fig. 7).

## Discussion

**Fig. 8:**
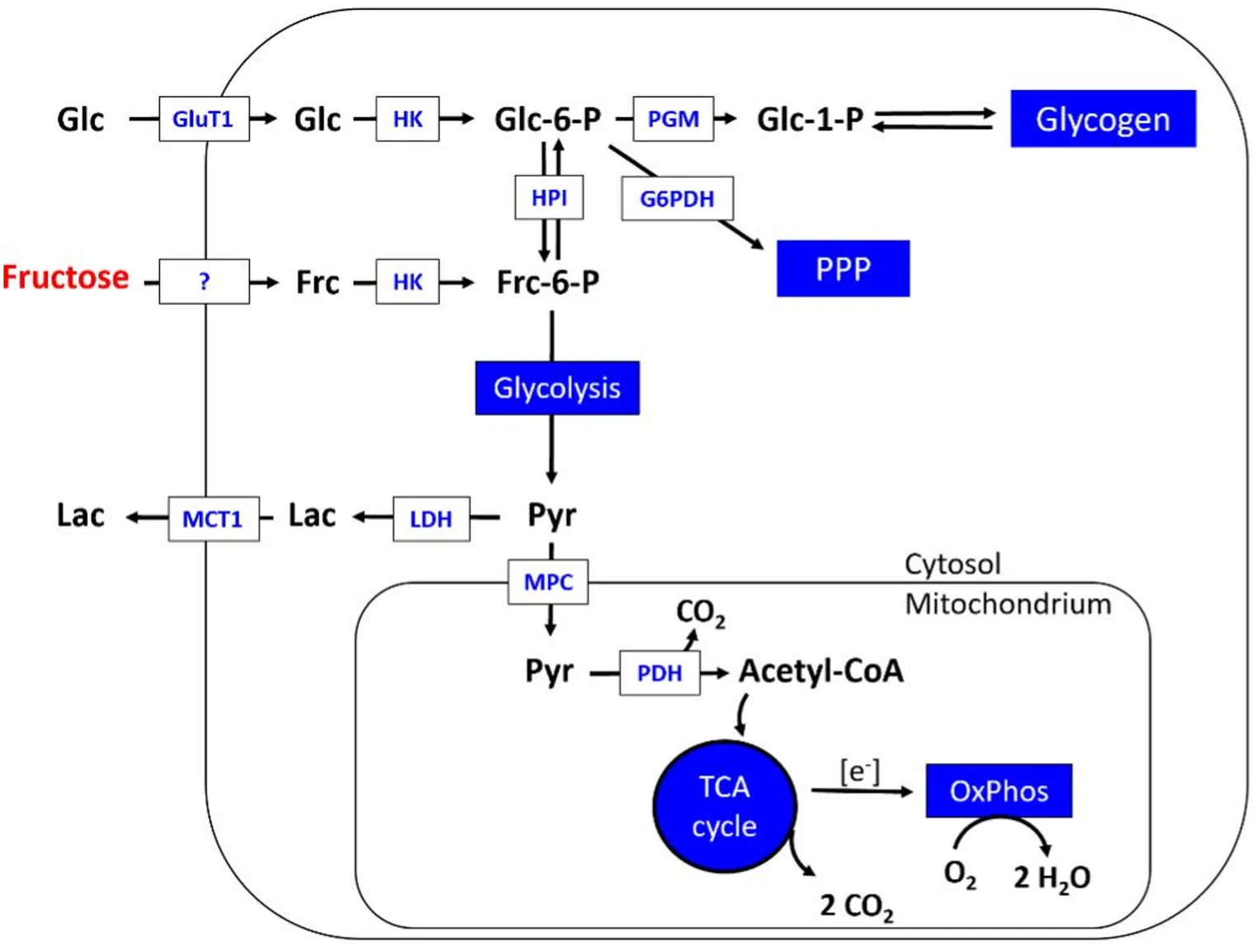
Pathways involved in the utilization of fructose for the metabolism of astrocytes. In astrocytes, both fructose and glucose are phosphorylated by hexokinase (HK). The two hexoses are taken up into the cells by different processes. Glucose is efficiently internalized by the GluT1 transporter, while the slower fructose uptake involves diffusion and/or GluT5-mediated transport. The HK products glucose-6-phosphate (Glc-6-P) and fructose-6-phosphate (Frc-6-P) are in an equilibrium that is established by hexose phosphate isomerase (HPI). Glc-6-P can be isomerized to glucose-1-phosphate (Glc-1-P) by the phosphoglucomutase (PGM) which connects the hexose phosphates with the glycogen metabolism. Glc-6-P can also enter the oxidizing part of the pentose phosphate pathway (PPP) via Glc-6-P dehydrogenase (G6PDH). Degradation of Frc-6-P via glycolysis leads to some ATP regeneration and to the formation of pyruvate (Pyr). This intermediate can be reduced to lactate (Lac) by the lactate dehydrogenase (LDH) and the formed lactate is released from astrocytes mainly via the monocarboxylate transporter 1 (MCT1). Alternatively, cytosolic pyruvate can be imported into mitochondria via the mitochondrial pyruvate carrier (MPC) and oxidatively decarboxylated via pyruvate dehydrogenase (PDH) to acetyl-Co that enters the tricarboxylic acid (TCA) cycle. The reduction equivalents [e^−^] obtained by the oxidoreductase reactions of the TCA cycle are used for effective ATP regeneration by the mitochondrial oxidative phosphorylation (OxPhos)

Fructose has frequently been shown to be used by astrocytes as exogenous substrate to fuel different metabolic processes (Fig. 8), including serving as carbon source for the synthesis of glycogen, lactate and pyruvate, as electron source for the reduction of peroxides and xenobiotics, and as exogenous substrate to maintain a high cellular ATP content during starvation (Table 1). Most of these studies report that fructose is – even if applied in an unphysiologically high concentration of 5 mM – less efficiently used by astrocytes than glucose. This is consistent with the observed rates of hexose consumption and lactate production which were found to be around 4 times higher for glucose compared to fructose.

Astrocytes showed a concentration-dependent linear fructose consumption that followed apparent Michaelis-Menten kinetics with a K_M_ value of 2.8 ± 0.9 mM and a V_Max_ value of 325 ± 32 nmol/(mg x h). Hyperbolic concentration-dependencies were previously reported for the astrocytic consumptions of the mitochondrial energy substrates pyruvate and proline with maximal consumption velocities of 300 nmol/(mg x h) for pyruvate [26] and 100 nmol/(mg x h) for proline [29], which are in a similar range to that found for fructose. The determined half-maximal consumption rates for pyruvate (0.6 mM) and proline (0.32 mM) were discussed to be in the concentration ranges that represent the physiological concentrations of these substrates in serum [26, 29]. In contrast, the concentration of fructose that allowed half-maximal consumption by astrocytes (2.8 mM) was orders of magnitude higher that the low micromolar concentrations of fructose found in serum [12–15].

Limiting for the utilization of fructose by astrocytes could be the uptake and/or the cellular phosphorylation of the internalized fructose. Cellular uptake of fructose in cells is mainly mediated by GluT5 [7, 8, 52, 53]. This transporter has a rather high K_M_ value for its substrate fructose which ranges between 6 and 12 mM [54–56]. GluT5 has been reported to be expressed in cultured mouse astrocytes [37, 57]. However, MSNBA which has been reported to selectively inhibit GluT5 without measurably affecting other GluT transporters [51] had only a partial inhibitory effect on fructose consumption and fructose-dependent lactate production by cultured rat astrocytes. The applied inhibitor concentration of 100 µM was far higher than the concentrations of MSNBA that have been reported to efficiently inhibit GluT5-mediated fructose uptake in human cancer cells with an IC_50_ value of 5.8 µM [51]. The low potential of a high concentration of MSNBA to affect astrocytic fructose consumption suggest that GluT5 may be involved to some extent in astrocytic fructose uptake, but that also other processes contribute to this uptake (Fig. 8). For example, diffusion has to be considered for astrocytic fructose uptake, as the concentration-dependency found for the uptake of radioactive fructose by astrocytes was linear up to a concentration of 50 mM [38]. Further studies, for example the generation of an astrocyte-specific knockdown of GluT5, would allow to investigate whether and to which extend GluT5 may contribute to fructose uptake and consumption by astrocytes. Nevertheless, uptake of fructose in the low millimolar concentration range by either GluT5 and/or by diffusion will strongly depend on the concentration of fructose applied.

The expression level of fructokinase is at best very low in cultured mouse astrocytes [58, 59] and the enzyme has not been detected in cultured rat astrocytes [30], suggesting that fructose metabolism in astrocytes is not initiated by phosphorylation of the hexose by fructokinase, but rather by HK-mediated generation of fructose-6-phosphate (Fig. 8). Analysis of the kinetic parameters of HK in astrocytic lysates for fructose and glucose revealed that both substrates were phosphorylated with similar V_Max_ values, but that the K_M_ value for fructose was with 4.4 ± 1.1 mM almost two orders of magnitude higher than that for glucose. Such severe differences between millimolar K_M_ values of HK for fructose and micromolar K_M_ values for glucose are consistent with literature data for HK from several cell types [43, 44, 60–62]. Thus, in addition to a slow uptake of fructose in astrocytes, the HK-mediated phosphorylation of fructose to fructose-6-phosphate could limit fructose metabolism in astrocytes.

The observation that hardly any free fructose was detectable in cultured astrocytes even in the presence of 10 mM of extracellular fructose, demonstrates that the cellular uptake of fructose limits its consumption rather than the phosphorylation, despite of the high K_M_ values of HK for fructose. Only if fructose concentrations higher than 10 mM were applied, free fructose was found in the cells, suggesting that for such conditions the rate of fructose uptake was higher than the rate of fructose phosphorylation. However, the specific content of free fructose was after exposure to 15 mM and 20 mM fructose only around 4.8 and 8.6 nmol/mg, respectively. These specific values represent cytosolic concentrations of 1.2 mM and 2.1 mM, as calculated by making use of the specific cytosolic volume of 4.1 µL/mg protein of astrocyte cultures [63]. Thus, cellular concentrations of free fructose account only to around 10% of the respective extracellular concentrations, supporting the view that predominantly a slow uptake of fructose limits its utilization for astrocytic metabolism and not the high K_M_ values of HK for fructose. A slow astrocytic uptake of fructose would also explain why astrocytes were reported to use 5 mM extracellular fructose less efficiently than 5 mM glucose at least for studies that have compared both hexoses for their potential to support glycolytic lactate formation, glycogen restoration or NADPH regeneration via the PPP (Table 1).

Fructose consumption by astrocytes involves uptake into the cells, phosphorylation by HK and further metabolism of the generated fructose-6-phosphate via glycolysis. Considering that for low millimolar concentrations the cellular uptake limits fructose consumption, the K_M_ values for the HK-mediated phosphorylation of cellular fructose and the K_M_ value for the overall consumption of extracellular fructose should be identical. Indeed, the values for cellular fructose consumption (2.8 ± 0.9 mM, *n* = 3; calculated from the linear decline of extracellular fructose concentrations during an incubation time of 16 h) and the K_M_ values for the phosphorylation of fructose by astrocytic HK (4.4 ± 1.1 mM, *n* = 4; determined for an incubation time of 20 min) did not significantly differ, supporting the view that fructose uptake and not cellular metabolism limits fructose metabolism in astrocytes.

For glucose-treated astrocytes, free cellular glucose was already detectable, if the cells had been incubated with extracellular glucose concentrations of above 5 mM, consistent with literature data [31], demonstrating that the glucose taken up during incubations with up to 5 mM is immediately phosphorylated by HK. Thus, for glucose in concentrations of up to 5 mM, the uptake into the cells and not the phosphorylation of glucose by HK limits glucose metabolism by astrocytes. The specific cellular contents of free glucose found after incubation with 10 mM, 15 mM and 20 mM of glucose corresponded to cytosolic concentrations of 4.3 ± 0.5 mM, 8.6 ± 0.8 mM and 12.0 ± 0.6 mM, respectively, which are in the same range as the concentrations of glucose applied. This supports the view that cultured astrocytes efficiently take up glucose under such condition and that for high glucose concentrations the transporters involved had within 1 h almost established an equilibrium between cellular and extracellular glucose concentrations.

Fructose has been shown to fully maintain cellular ATP levels in cultured astrocytes during starvation for 24 h [27]. Analysis of the concentration dependencies of the ATP maintenance for fructose and glucose revealed that both hexoses have a similar potential to prevent in cultured astrocytes the loss in cellular ATP during starvation. Already 1 mM of fructose or glucose was able to fuel ATP regeneration and to maintain a high cellular ATP content for 24 h. As no extracellular lactate was detectable after such an incubation, it can be assumed that the applied hexoses had been fully metabolized and that also all the lactate that was generated from the hexoses and released during the incubation had been taken up and fully oxidized by mitochondrial processes. Thus, for such conditions, astrocytes appear to make use of their potential to completely oxidize the carbon provided to fuel ATP regeneration, irrespective of whether the available carbon source was provided as fructose or glucose. This suggests that mitochondrial oxidative phosphorylation is crucial for the utilization of fructose as energy source by astrocytes.

For glucose-fed astrocytes, an impairment of mitochondrial respiration causes a strong upregulation of glycolytic flux [32, 47] which helps to prevent a rapid decline in cellular ATP regeneration [27]. Also for fructose-fed astrocytes lactate production was almost doubled in the presence of inhibitors of mitochondrial respiration, but this upregulation of fructose-driven glycolysis did not even reach the basal lactate release found for glucose-fed astrocytes in the absence of mitochondrial inhibitors. Thus, astrocytic fructose catabolism cannot be upregulated after inhibition of mitochondrial oxidative phosphorylation to an extent that will allow the maintenance of a high ATP level by accelerated glycolysis and prevent toxicity.

The initial specific rates of cellular consumption and metabolism differed strongly for fructose- and glucose-fed astrocytes, reflecting the different potential of the cells to take up and phosphorylate these hexoses. However, comparison of the consumption of hexose-derived carbon revealed that similar amounts of around 10 mM extracellular carbon had been consumed by the cells within the 24 h exposure to fructose and glucose, which are likely to reflect predominantly the mitochondrial oxidation of the hexose carbon to CO_2_. The ability of cultured astrocytes to oxidize fructose-derived carbon for mitochondrial ATP regeneration (Fig. 8) explains, why fructose has an almost identical potential as glucose to maintain a high ATP content in starved astrocytes, while fructose is much less potent than glucose to fuel other pathways in cultured astrocytes (Table 1).

Accordingly, inhibition of mitochondrial oxidative phosphorylation severely impaired the ability of astrocytes to use fructose as extracellular energy substrate which caused rapid and complete loss in cellular ATP content and subsequently severe cell damage that is known as consequence of a severe ATP depletion [27, 64]. In contrast, for glucose-treated astrocytes, the strongly accelerated glucose consumption and lactate production found for conditions that impair mitochondrial respiration [32, 47], prevented a rapid loss in cellular ATP content and cell damage as long as extracellular glucose was available. These data clearly demonstrate that astrocytes need a functional mitochondrial oxidative phosphorylation for using fructose as exogenous energy substrate.

The astrocytic consumption of both fructose and glucose as well as the lactate formation during incubation with theses hexoses were significantly lowered by the presence of 2DG. This was expected as 2DG competes with fructose or glucose for phosphorylation by HK and has been shown to lower lactate production from fructose and glucose [30, 42]. However, such a treatment with 2DG did only partially lower cellular ATP content during incubations with fructose or glucose which is likely to be caused by the initial rapid phosphorylation of 2DG to 2DG-6-phosphate by HK, while a rapid ATP loss and cell toxicity were prevented by ATP regeneration via mitochondrial oxidation of endogenous substrates such as fatty acids [27, 42].

In conclusion, the data presented in this study demonstrate that millimolar concentrations of fructose enable cultured astrocytes to metabolize this hexose as energy substrate and that oxidative phosphorylation of fructose-derived metabolites is essential for this process. Consumption and metabolism of fructose depend strongly on the available fructose concentration and are limited by a slow uptake into the cells. Thus, if applied in millimolar concentrations at least cultured astrocytes can utilize and metabolize fructose as energy substrate. Whether astrocytes in brain do substantially contribute to the fructose metabolism of brain can be doubted, at least for physiological conditions, as fructose is present only in low micromolar concentrations in serum [12–15]. Further studies are now required to investigate whether and how an adaptation of astrocytes to higher concentrations of fructose may alter the potential of these cells to metabolize fructose.

Excessive consumption of fructose has been connected with alterations in brain function, including neuroinflammation, mitochondrial disfunctions, alterations in synaptic plasticity and cognition as well as impairment in learning and memory [16–19]. Whether astrocytes play a role in such adverse processes remains to be investigated, but appears unlikely as the astrocytic fructose uptake and metabolism is not very efficient at the low concentrations of blood-derived fructose [12–15], as fructose in concentrations as high as 20 mM did not have any obvious adverse effects on cultured astrocytes, and as the astrocytic fructose metabolism in vivo would compete with the normal glucose metabolism. In contrast to astrocytes, several other brain cells types appear to be affected by elevated fructose concentrations. Short-term fructose ingestion has been shown to lower hippocampal myelin basic protein content, pointing to a possible sensitivity of oligodendrocytes or myelination to fructose exposure [37]. In addition, single-cell transcriptomic analysis have identified additional brain cell populations, including neurons, that respond to dietary fructose [65] and fructose exposure has been connected to reductions in synaptic markers, suggesting adverse effects on synaptic integrity [66, 67]. Also the upregulation of the fructokinase-dependent pathway for fructose metabolism in microglia has been linked to mitochondrial damage and cognitive dysfunction in a mouse model for diabetes [68], while early-life exposure to high dietary fructose has been reported to impair microglial phagocytic function and neurodevelopment [69]. A poor ability of astrocytes in brain to take up and metabolize fructose that is entering the brain from the blood, even under conditions of elevated fructose concentrations, may allow this hexose to further permeate into the brain, thereby reaching other brain cells that are more susceptible toward the adverse effects of an excess of fructose.

## Supplementary Information

Below is the link to the electronic supplementary material.

Supplementary material 1 (PDF 514.1 kb)

## Data Availability

Enquiries on original data should be directed to the corresponding author.
