# Supplementary material for: Consumption of Fructose by Cultured Primary Rat Astrocytes
Source: Neurochem Res. 2026 Aug 27;51(5):253. doi: 10.1007/s11064-026-04873-w (PMC13522079; doi:10.1007/s11064-026-04873-w)
Supplement: Supplementary file 1 [file 11064_2026_4873_MOESM1_ESM.pdf]

Consumption of fructose by cultured primary rat astrocytes

Mariana Mesa-Leal and Ralf Dringen

Supplementary Information

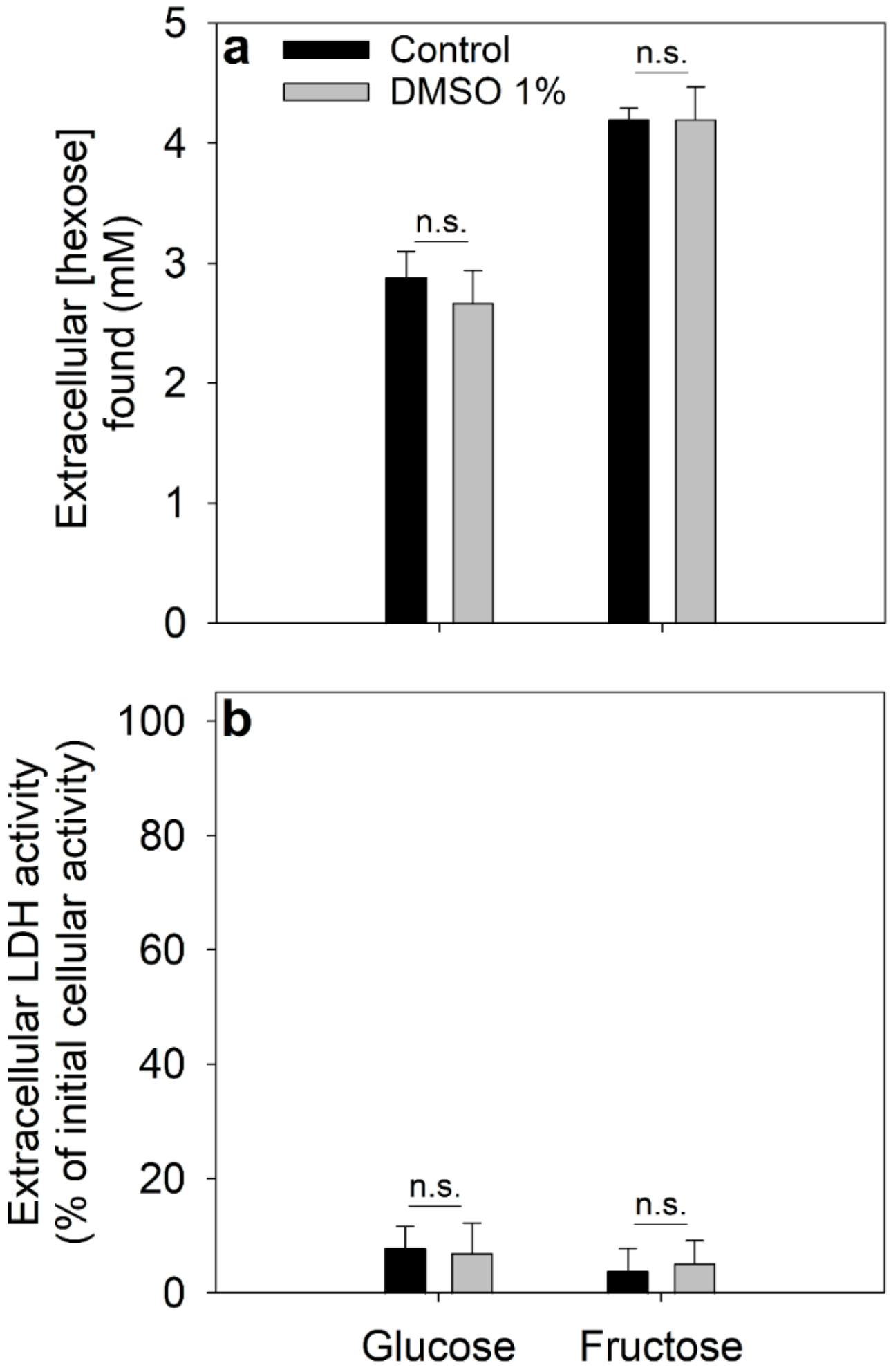

Figure S1: Test for the effects of the solvent DMSO on the consumption of glucose or fructose by cultured astrocytes. Cultured primary astrocytes were incubated for 6 h with 5 mM glucose or fructose in the absence or the presence of 1% (v/v) DMSO before the extracellular hexose concentrations (a) and the extracellular LDH activities (b) were determined. The initial cellular LDH activity was 103 ± 5 nmol (min × well) and the initial cellular protein content was 111 ± 19 µg/well. The data represent the means ± SD from three independent experiments, performed on independently prepared cultures. Statistical analysis of the date by a paired t-test revealed no significant difference (n.s.) between the data obtained for control and DMSO-treated cells.

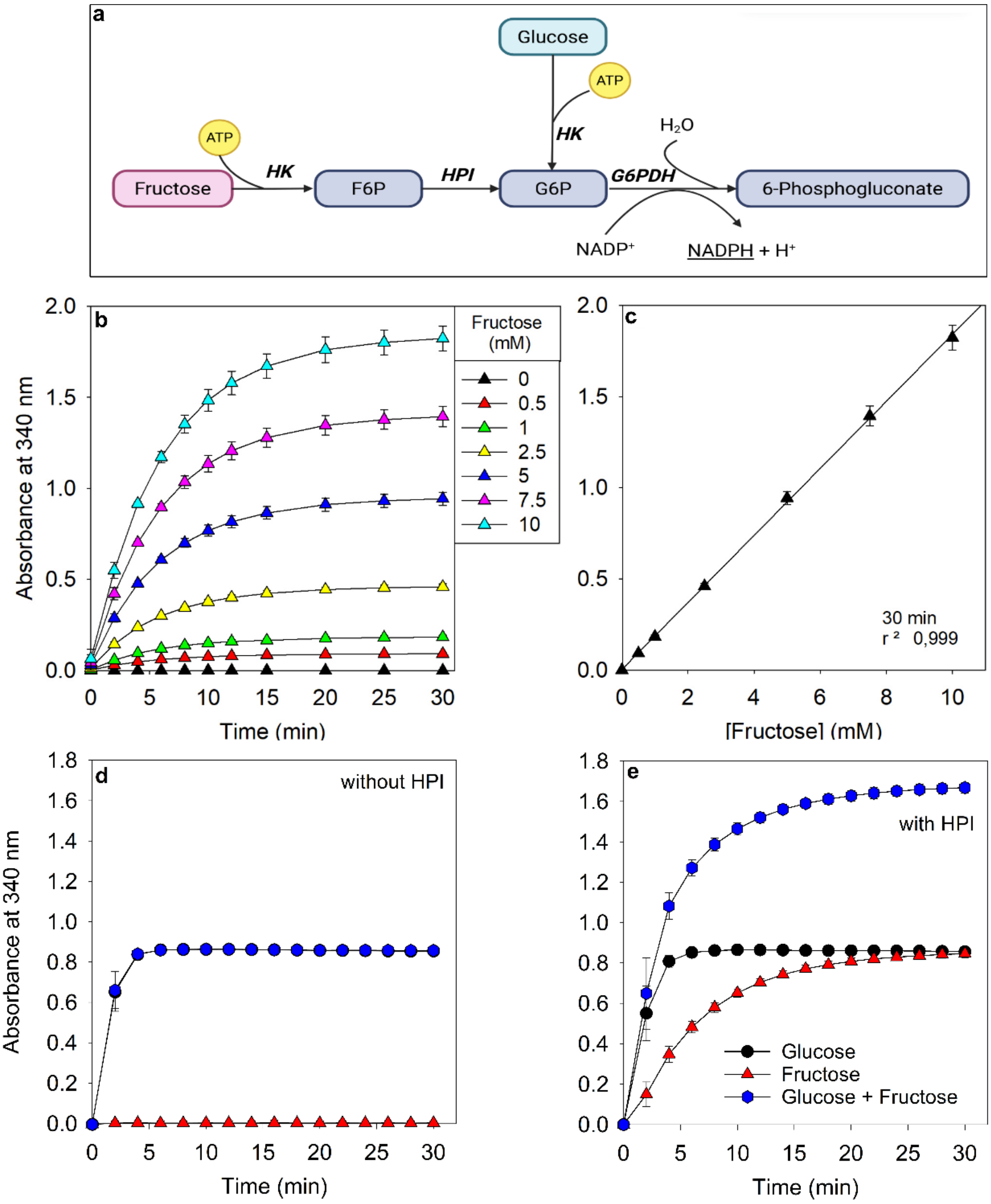

Figure S2: Quantification of fructose by coupled enzymatic assays in the absence or presence of glucose. Assay principle: fructose is phosphorylated to fructose-6-phosphate (F6P) by hexokinase (HK). F6P is then isomerized to glucose-6-phosphate (G6P) by hexose phosphate isomerase (HPI). Finally, G6P is oxidized by the $NADP^+$-dependent enzyme glucose-6-phosphate dehydrogenase (G6PDH) to 6-phosphogluconate. The resulting NADPH is measured by recording its absorbance at 340 nm. Glucose is detected via the couple reaction involving HK and G6PDH. In the absence of PGI, only glucose is measured, while the presence of PGI allows for the quantification of the combined amounts of glucose and fructose (a). The time course of NADPH formation was measured for a 10 µL sample volume of the given fructose concentrations in glucose-free incubation buffer (b). Calibration curve correlating the applied fructose concentration with the absorbance at 340 nm determined after 30 min of reaction time (c). Increase in absorbance over time for reactions containing 5 mM glucose and 5 mM fructose in the absence (d) or the presence (e) of HPI. The data shown in panels b to e are means ± SD of values obtained in three independent experiments.

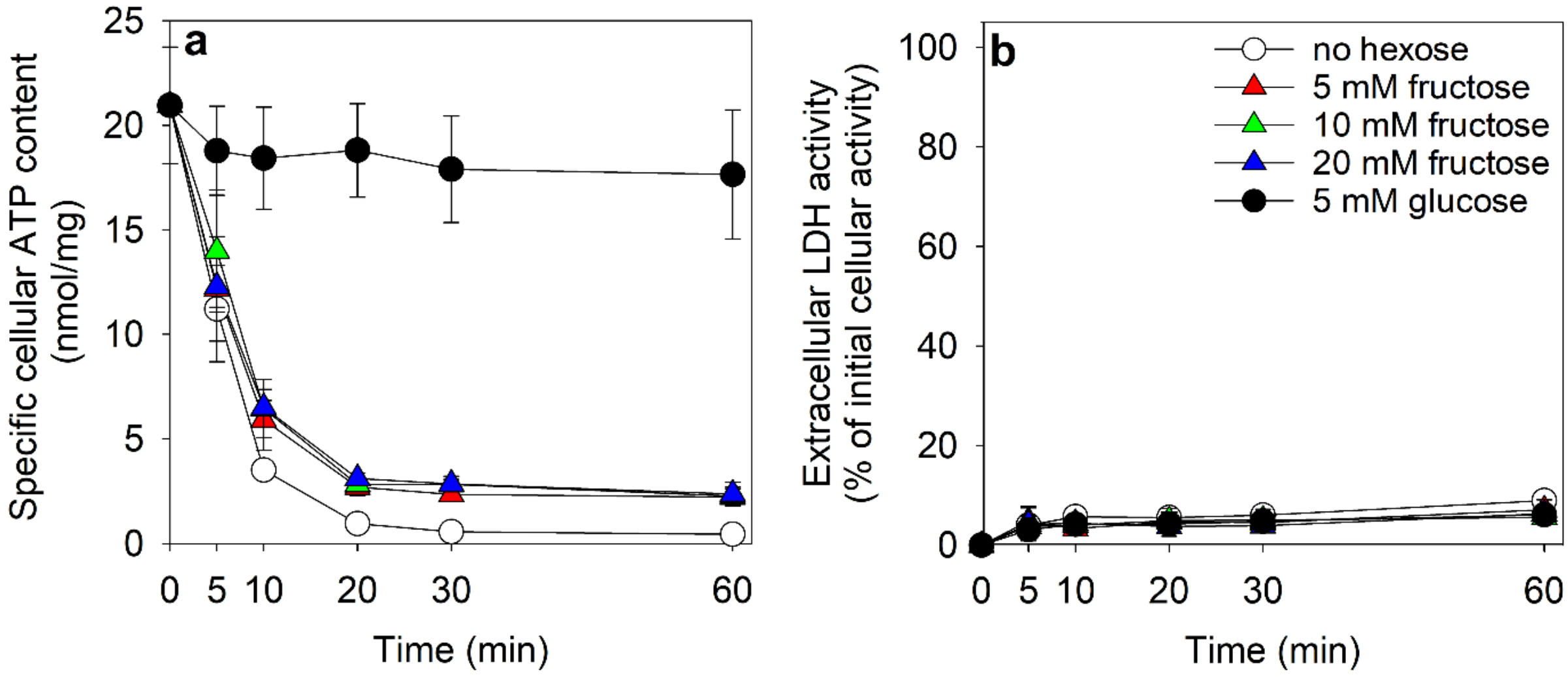

Figure S3. ATP maintenance by fructose or glucose in antimycin A-treated cultured astrocytes. The cultures were incubated for up to 60 min in glucose-free incubation buffer with 1 µM antimycin A in the absence (none) or the presence of fructose or glucose in the given initial concentrations before the specific cellular ATP content (a) and the extracellular LDH activities (b) were determined. The initial cellular LDH activity was 108 ± 3 nmol/(min x well) and the initial protein content was 175 ± 9 µg/well. The data represent the means ± SD from three independent experiments, performed on independently prepared cultures.
